# Comparative Transcriptional Analysis of Long Noncoding RNAs in Oxidative Stress and Inflammation Induced by Potassium Permanganate and Lipopolysaccharide in Rat Uterine Tissues

**DOI:** 10.3390/antiox14030251

**Published:** 2025-02-21

**Authors:** Talha Umar, Huili Feng, Wen Feng, Han Zhou, Nuoer Chen, Jinxin Zhang, Wenjing Liu, Xiao Wang, Saqib Umer, Zaima Umar, Muhammad Asad, Muhammad Naeem, Changwei Qiu, Ganzhen Deng

**Affiliations:** 1 Department of Clinical Veterinary Medicine, College of Veterinary Medicine, Huazhong Agricultural University, Wuhan 430070, China; talhaumar@webmail.hzau.edu.cn (T.U.); sdqiu2001@mail.hzau.edu.cn (C.Q.); 2 Department of Theriogenology, Faculty of Veterinary Science, University of Agriculture, Faisalabad 38000, Pakistan; saqib.umer@uaf.edu.pk; 3 Department of Anatomy, The University of Faisalabad, Faisalabad 38000, Pakistan; zaima.umar@tuf.edu.pk; 4 National Key Laboratory of Agricultural Microbiology, College of Veterinary Medicine, Huazhong Agricultural University, Wuhan 430070, China; 5 Department of Veterinary Medical Sciences, University of Parma, I-43100 Parma, Italy

**Keywords:** potassium permanganate, lipopolysaccharides, oxidative stress, inflammation, toxicity, LncRNAs, mRNAs

## Abstract

Potassium permanganate (KMnO_4_) is a commercially available antiseptic used in bovine intrauterine lavage to manage postpartum infections. Lipopolysaccharides (LPS) are well-studied for their ability to induce inflammation and oxidative stress. While KMnO_4_ is known to cause significant irritation, oxidative stress, and toxicity in uterine tissues, its transcriptional impact and potential for inducing similar molecular damage as LPS have not been fully explored. In this study, we induced oxidative stress in the uterine tissues of Sprague–Dawley (SD) rats using KMnO_4_ and compared the transcriptional profiles with those treated with LPS. We focused on the differential expression of long noncoding RNAs (lncRNAs) and messenger RNAs (mRNAs) related to oxidative stress, toxicity, and inflammation. RNA sequencing revealed 1125 differentially expressed mRNAs in the KMnO_4_-treated group and 989 in the LPS-treated group. Additionally, 1649 lncRNAs were differentially expressed in the KMnO_4_ group compared with 1383 in the LPS group. Gene ontology (GO) and KEGG enrichment analyses showed that 78 pathways were significantly enriched in the KMnO_4_ group, while 80 pathways were enriched in the LPS group, with 50 pathways shared between the two. This study offers critical insights into the transcriptional profiles associated with KMnO_4_ exposure and its similarities to LPS-induced damage.

## 1. Introduction

Postpartum infections persist as a significant challenge in obstetrics, even though advancements in hygiene standards and enhanced diagnosis and treatment tools have led to a decrease in maternal mortality from infections in developed countries [1]. During parturition, the uterus can become contaminated with bacteria, leading to postpartum uterine infections such as pruritis, metritis, and endometritis [2]. The incidence of uterine infections exhibits significant variation among different research studies. Uterine infection is characterized by the attachment of infectious microorganisms to the lining of the uterus, their growth and spread within the tissue, and the production of bacterial toxins, all of which contribute to the development of uterine disease [3,4].

Previous research has demonstrated the recovery of bacteria from the uterine tissues of infected cows, mainly identified as *Escherichia coli* (*E. coli*), *Staphylococcus aureus* (*S. aureus*), and *Trueperella pyogenes* (*T. pyogenes*) [2,5]. *E. coli* is the most prevalent gram-negative bacterium among all mentioned microorganisms, leading to uterus infection through LPS. It is also used to design many inflammatory models in animals and cells [6,7]

Potassium permanganate (KMnO_4_) is a potent oxidizing agent with strong antiseptic properties. It has been traditionally used for uterine lavage in cows, especially in cases of postpartum infections such as metritis and endometritis [8]. While it is effective as a disinfectant, KMnO_4_ is also known to cause tissue irritation and oxidative stress, leading to ulcerative injury and apoptosis [9]. Despite these adverse effects, it remains in use in various regions because of its accessibility and antimicrobial properties [10,11]; however, the exact molecular damage caused by KMnO_4_ in uterine tissues has not been fully explored, particularly in comparison to the well-established inflammatory responses induced by LPS.

RNA molecules longer than 200 nucleotides in length that do not code for proteins but play critical roles in gene expression at both the transcriptional and post-transcriptional levels are called long noncoding RNAs (lncRNAs) [12,13]. Research has demonstrated that lncRNAs regulate numerous biological processes, including inflammation, oxidative stress, cell differentiation, apoptosis, and reproductive functions [14,15]. In the female reproductive system, specific lncRNAs have been shown to modulate critical processes, such as folliculogenesis, spermatogenesis, and embryo implantation [16,17]. For example, NEAT1 has been linked to corpus luteum formation and fertility [18], while lncRNA-H19 has been implicated in regulating endometriosis [19,20]. These findings highlight the crucial regulatory roles that lncRNAs play in uterine biology and disease.

Given that LPS and KMnO_4_ are both associated with oxidative stress and inflammatory responses in uterine tissues, understanding their transcriptional effects is essential. This study aims to compare the transcriptional profiles of rat uterine tissues exposed to KMnO_4_ and LPS, focusing on the expression of lncRNAs and mRNAs involved in oxidative stress, toxicity, and inflammation. For the first time, we hypothesize that KMnO_4_ induces molecular damage similar to that caused by LPS, with both agents activating overlapping pathways related to oxidative stress and inflammation. Additionally, this study seeks to identify potential interactions between lncRNAs and their target mRNAs, paving the way for future research into the regulatory roles of lncRNAs in uterine health.

## 2. Material and Methods

### 2.1. Animal Model

The Animal Experiment Centre of Huazhong Agricultural University (Wuhan, China) provided eighteen adult female Sprague–Dawley (SD) rats weighing between 190–200 g. The rats were housed under standard conditions with a 12-h light/dark cycle, fed a standard diet, and provided with water ad libitum for an acclimatization period of 7 days. After acclimatization, the rats were randomly assigned to three groups (*n* = 6 per group): a control group, a potassium permanganate (KMnO_4_) group, and a lipopolysaccharide (LPS) group. The KMnO_4_ used in this experiment was purchased locally, and a 0.05% solution was prepared by dissolving the powder in distilled water. For dosing, each rat’s body weight was used to calculate the appropriate dose of KMnO_4_ and LPS. A dose of 10 mg/kg body weight was administered to the KMnO_4_ group. Given that the rats weighed approximately 200 g, the dose per rat was calculated as follows:

Dose per rat = Body weight (kg) × Dose (mg/kg) = 0.2 kg × 10 mg/kg = 2 mg

The 2 mg of KMnO_4_ was diluted in distilled water and delivered as an intrauterine injection with a total volume of 20 µL. The LPS group received an intrauterine injection of LPS (50 µL of a 1 mg/mL solution), equivalent to 50 µg of LPS per rat. The control group received an intrauterine instillation of 50 µL of normal saline. Specimens from all groups were collected 48 h after injection for histopathological analysis to confirm the induction of tissue damage and inflammation, validating the success of the experimental model.

### 2.2. Histopathology and Wet and Dry Weight (W/D)

Uterine tissue in the fixative solution was removed and rinsed with water for 30 min, dehydrated in a vacuum tissue dehydrator using 30%, 50%, and 75% ethanol for 2 h, 85%, 90%, and 95% for 1.5 h, and anhydrous ethanol twice for 1 h each time. Transparent xylene was twice for 20 min each, and molten paraffin was soaked twice for 1 h each. For embedding, the uterine tissue was removed from the dehydrant and put into the embedding frame. The molten paraffin was poured into the frame, and the wax block was removed and trimmed after the wax solidified and sliced at a thickness of 5 μm. The transparent slices were rehydrated and washed with tap water. Hematoxylin & eosin (H&E) staining was observed under electron microscopy. In order to determine the wet and dry weights of the rat uterine tissues, the tissues were surgically removed and then rinsed with PBS three times. Subsequently, they were placed in an incubator at a temperature of 80 °C for 24 h. Following this 24 h period, we determined the uterus tissues’ wet-to-dry ratio (W/D).

### 2.3. Myeloperoxidase (MPO), Malondialdehyde (MDA), and Superoxide Dismutase (SOD) Assays

An appropriate amount of uterine tissue was cut, weighed, and ground in liquid nitrogen until crushed into a fine powder. An isotonic sodium chloride solution was added according to mass:volume = 1:9 to obtain a 10% tissue homogenate that was centrifuged at 4 °C, 13,000× *g* for 10 min. The supernatant was collected, and the MPO and MDA content and activity of SOD were measured (Nanjing Jiancheng Bioengineering Institute, Nanjing, China). The testing steps strictly followed the kit instructions.

### 2.4. RNA Extraction and cDNA Library Construction

After retrieving the cryopreservation tube containing the uterine tissues, it was placed on ice for 5 min. Next, approximately 30 mg of uterine tissue was carefully excised using surgical scissors. Subsequently, the excised tissue was placed into a centrifuge tube with a volume capacity of 2 mL. A volume of 1 mL of Trizol reagent was added to the tube containing the uterine tissue. The extraction of total RNA from the control, LPS, and KMnO_4_-treated groups, with two samples per group, was performed using RNAiso Plus (Takara, Dalian, China) following the guidelines provided by the manufacturer. The utilization of total RNA serves as the initial material for the construction of a complementary DNA (cDNA) library. RNA purification, reverse transcription-based cDNA synthesis, adaptor ligation and end repair, PCR amplification, and library construction are the main processes in this procedure. The library was sequenced using the Illumina HiSeq 4000 platform, made in San Diego, CA, USA, by Illumina Inc., after quality inspection and preparation.

### 2.5. Quality Control Data of Sequencing

After the base recognition process, the initial image file (BCL) received by sequencing was transformed into raw data in the FASTQ format. The initial dataset was evaluated comprehensively to determine its suitability for bioinformatics analysis. The primary components of the quality analysis encompassed the evaluation of sequencing quality and the investigation of base composition—the acquisition of clean data involved eliminating linker and low-quality sequences from raw data. The evaluation of data cleanliness was subsequently conducted using FASTQC v0.10.1.

### 2.6. The Alignment of a Reference Genome and the Mapping of Read Distribution Across the Entire Genome

Clean readings were obtained using Trimmomatic (v3.0) after these data were filtered, and ribosomal RNA was eliminated using Bowtie2 (v2.2.5). HISAT2 (v2.1.0) was used to align the paired-end clean reads to the reference genome, and Bowtie2 (v2.2.5) was used to create the reference genome index. Using a reference-based methodology, the Scripture (beta2) and Cufflinks (v2.1.1) software produced the assembled transcripts for every sample [21]. Furthermore, each sample’s aligned reads were created using a reference-based method with StringTie (v1.3.1) [22].

### 2.7. Analyzing the Differential Expression

HTSeq (v0.11.2) was used for the quantification of genes and transcripts. To find differential expressions in these transcriptomic or genomic data, DESeq2 (v1.18.1) was utilized. The thresholds for identifying significant differential expression in abiotic replication are an absolute value of the logarithm base 2 of the fold change <1, and it has been determined that transcripts or genes with a *p* < 0.05 indicate differential expression in biological replication.

### 2.8. LncRNA-Gene Interaction Predictions

The mRNA and lncRNA transcripts were screened, followed by quantitative analysis using the StringTie-eB program. This analysis determined transcript abundance, namely the FPKM values, for each sample transcript. In the previous study, the authors employed Cuffdiff (version 2.1.1) to compute the Fragments Per Kilobase of transcript per Million mapped reads (FPKM) for both long non-coding RNAs (lncRNAs) and coding genes across all samples [23]. Transcripts with an adjusted *p*-value of less than 0.05 were classified as differentially expressed.

### 2.9. KEGG and GO-Based Functional Annotation and Enrichment Analysis

The GOseq R package (v.1.58.0) conducted GO enrichment scores on differentially expressed genes and lncRNA target genes. Significantly enriched GO keywords were those with adjusted *p*-values below 0.05 [24]. The KEGG database is valuable for comprehending biological systems’ underlying functions and applications. It offers insights into several aspects, including cells, animals, and ecosystems, by leveraging molecular-level data, particularly about large-scale molecules. The genome sequencing and other high-throughput experimental methods used to collect the dataset used in this investigation are described in the following source: http://www.genome.jp/kegg/ (accessed on 15 January 2025). Using the KOBAS software (v.3.0.3), the enrichment analysis of lncRNA target genes or differentially expressed genes was carried out. [25].

### 2.10. RT-qPCR Validation

The RNA samples extracted from uterine tissues, including KMnO_4_-treated, LPS-treated, and untreated tissues, were analyzed using reverse transcription-quantitative polymerase chain reaction (RT-qPCR) for lncRNA and mRNA sequencing. Subsequently, complementary DNA (cDNA) was synthesized using a reverse transcriptase reagent following the guidelines provided in the instruction manual. The SYBR Green Plus Reagent Kit with the Light Cycler 96 equipment from Roche, Basel, Switzerland, was used to conduct a quantitative polymerase chain reaction (qPCR). This experiment adhered to the manufacturer’s recommendations, using GAPDH as the internal reference gene. The primer sequences employed in the investigation are shown in Table 1.

### 2.11. Statistical Analysis

This study will use statistical tools to analyze and interpret acquired data. The statistical studies were conducted utilizing the GraphPad Prism 9 software. A one-way analysis of variance (ANOVA) was used to assess if there were any significant variations in the levels of long non-coding RNAs (lncRNAs) and messenger RNAs (mRNAs) among the groups that were treated with LPS and KMnO_4_ as well as the control groups. Statistically significant differences were seen when the *p*-values were below the thresholds of 0.05 and 0.01, respectively.

## 3. Results

### 3.1. Histopathological Results of Uterine Tissues

**Figure 1:**
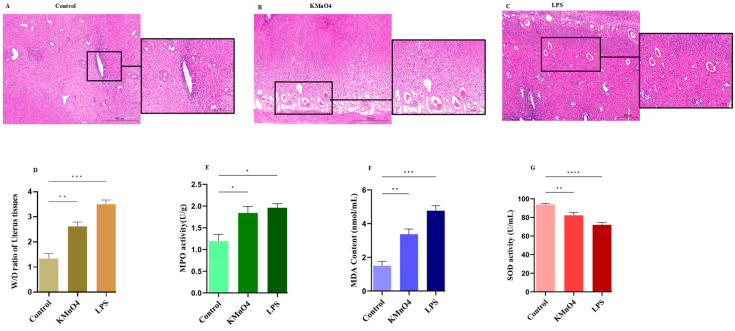
Histopathological analysis of uterine tissues. (**A**–**C**) Histopathological changes demonstrated by H&E staining: (**A**) Control group, (**B**) KMnO_4_ group, and (**C**) LPS group. Scale bar 200 μm. (**D**) Wet-to-dry ratio of uterine tissues in different groups. (**E**) Myeloperoxidase (MPO) assay results. (**F**) Malondialdehyde (MDA) activity. (**G**) Superoxide dismutase (SOD) activities. All data were measured as mean ± SEM by three independent experiments * *p* < 0.05, ** *p* < 0.01, *** *p* < 0.001, **** *p* < 0.0001. H&E Hematoxylin & Eosin; W/D Wet and Dry weight.

The uterus of SD rats was retrieved and treated with H&E staining. The H&E staining results proved that the control group showed no obvious alterations; however, the uterus treated in the KMnO_4_ and LPS groups displayed evident indications of edema, endometrial damage, cellular infiltrations, chromatin condensation, and necrosis (Figure 1A–C). The wet and dry ratios of the normal and KMnO_4_ and LPS groups were assessed by weighing tissues. The treatment groups exhibited significantly higher ratios than the normal group (*p* < 0.01; Figure 1D). MPO serves as both a function and activation marker for neutrophils and macrophages. It can be used to forecast the onset of early inflammatory illnesses by assessing the activity level of MPO (Li et al., 2015 [26]). The MPO assay was used to assess the infiltration of inflammatory cells, and the results revealed a significant increase in the treatment groups (*p* < 0.01; Figure 1E). Additional SOD and MDA key markers were employed to examine the oxidative stress in rats. Interestingly, KMnO_4_ and LPS substantially reduced SOD activity (Figure 1F) and increased MDA content (Figure 1G). These results demonstrate the successful establishment of our model.

### 3.2. Sequencing Data Statistics of Uterine Tissues

The present investigation involved the construction of a total of six complementary DNA (cDNA) libraries. This was achieved by extracting total RNA from uterine tissues obtained from KMnO_4_-treated, LPS-treated, and control samples. The cDNA library of various samples consisted of the following raw reads: control_1 with a count of 65,468,884, control_2 with a count of 60,513,980, and KMnO_4__1 with a count of 64,455,606, and KMnO_4__2 with a count of 59,971,680 and LPS_1 732,478,86, LPS_2 87,839,856. The GC content percentages were determined to be as follows: cont1, 50.55%; cont2, 51.83%, and KMnO_4__1, 49.93%; KMnO_4__2, 51.77% and LPS_1 49.87% and LPS_2 49.64%. These raw data were subjected to a filtering process to obtain clean data. Table 2 presents the counts of clean reads based on various parameters such as GC% and mapped reads (%) and their effective rate (%). Furthermore, the clean reads were matched to the rat reference genome using the HISAT2 program, which utilizes an enhanced Burrows–Wheeler Transform (BWT) technique known as the FM index. This analysis revealed that the total number of mapped reads or fragments across all samples exceeded 80%, and they were successfully aligned to the reference genome.

### 3.3. Chromosomal Distribution of Differentially Expressed Transcripts

**Figure 2:**
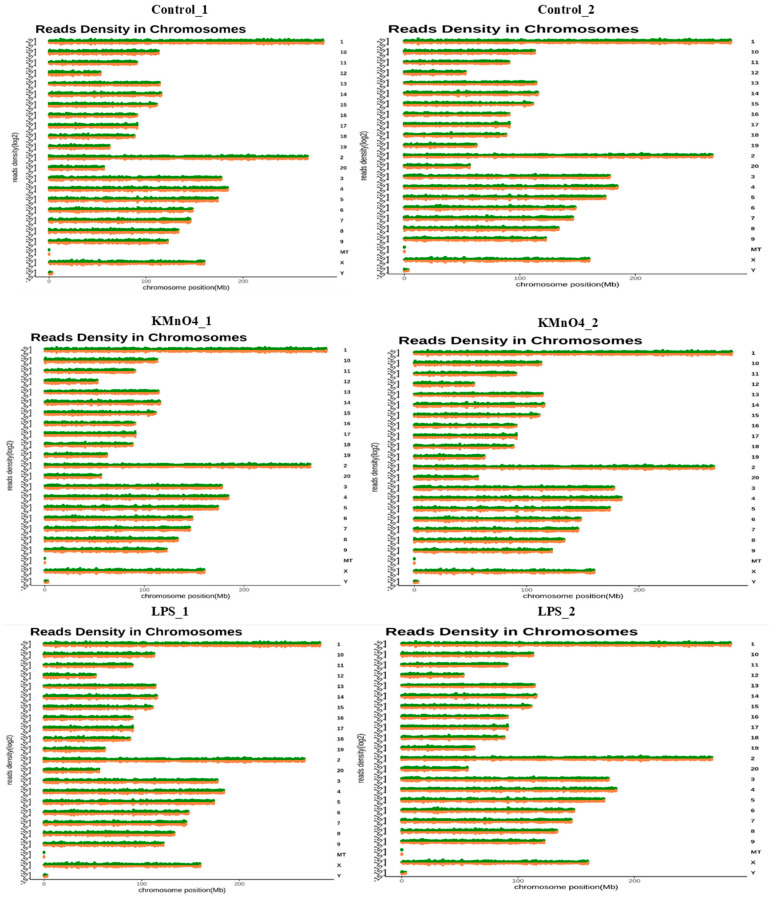
Chromosomal Distribution of Differentially Expressed Transcripts. This figure represents the chromosomal distribution of differentially expressed transcripts identified across all experimental groups. Control group (Control_1; Control_2); KMnO_4_ group (KMnO_4__1; KMnO_4__2); LPS-group (LPS_1; LPS_2).

In some cases, genes may have a discernible pattern of consistent arrangement along chromosomes, wherein genes located close to one other on a chromosome often demonstrate similarities in their biological functions or participation in the same metabolic pathway. The likelihood of gene regulation is higher for genes close to each other than for genes situated at greater distances; therefore, the distribution of distinct transcripts on chromosomes and the simultaneous differential expression of adjacent transcripts may have critical biological ramifications. Furthermore, this phenomenon facilitates the precise identification of genes pertinent to this study’s objectives. Figure 2 illustrates the specific distribution of differential transcripts across chromosomes. The homogeneous distribution of differentially expressed messenger RNAs (mRNAs) was seen across all chromosomes. The mRNAs that showed upregulation and downregulation in the control group, the KMnO_4_-treated and LPS groups were found on chromosomes 1, 2, 3, 4, 5, and X. The long non-coding RNAs (lncRNAs) that demonstrated an increase and decrease in expression mainly were situated on chromosomes 2, 3, 6, and 9.

### 3.4. Differentially Expressed Gene Analysis in KMnO_4_ Treated, LPS Treated, and Normal Uterine Tissues

**Figure 3:**
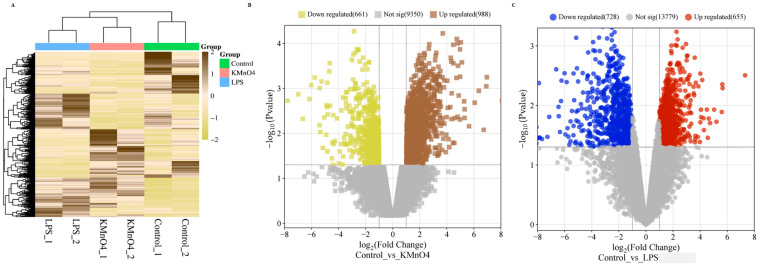
Analysis of Differentially Expressed Genes in KMnO_4_-Treated, LPS-Treated, and Normal Uterine Tissues. (**A**) Heatmap displaying hierarchical clustering of differentially expressed genes across the experimental groups based on FPKM values. Log10(FPKM + 1) transformation is applied for clustering. Genes with higher expression levels are represented in brown, while genes with lower expression are shown in yellow, indicating distinct expression profiles among the groups. (**B**) Volcano Plot of Differentially Expressed Genes in Control vs. KMnO_4_ Group illustrating the distribution of differentially expressed genes between the control and KMnO_4_-treated groups. Genes with significantly higher expression in the KMnO_4_ group are shown in brown, and genes with lower expression are shown in yellow. (**C**) Volcano Plot of Differentially Expressed Genes in LPS-Treated Group representing the differentially expressed genes in the LPS-treated group compared with the controls. Red dots indicate genes with significantly higher expression, while blue dots represent genes with lower expression, emphasizing the transcriptional shifts induced by LPS treatment.

The DESeq2 package was utilized to perform differential expression analysis on genes. The differentially expressed genes between samples were determined based on two criteria: a minimum fold change in ∣log2 Fold Change∣ > 1 and a significance level of *p*-value < 0.05 [27,28]. The expression levels of the differential genes were quantified using the RPKM/COUNT metric. The present work demonstrates the utilization of hierarchical clustering analysis to examine the patterns of differentially expressed genes, as depicted in Figure 3A. The KMnO_4_ and LPS groups showed a distinct expression pattern compared with the control group. There was no distinct difference between the KMnO_4_ and LPS groups.

The upregulated and downregulated genes were subjected to volcano plots. Data on the number of genes that exhibited differential expressions in the KMnO_4_-treated, LPS-treated, and control groups were presented in Figure 3B,C. A comprehensive analysis revealed 988 genes exhibiting considerable upregulation and 661 genes displaying significant downregulation in the control vs. KMnO_4_ group. In the control vs. LPS group, 655 were upregulated genes, and 728 were downregulated.

### 3.5. GO Functional Analysis of Differentially Expressed mRNA Enrichment

**Figure 4:**
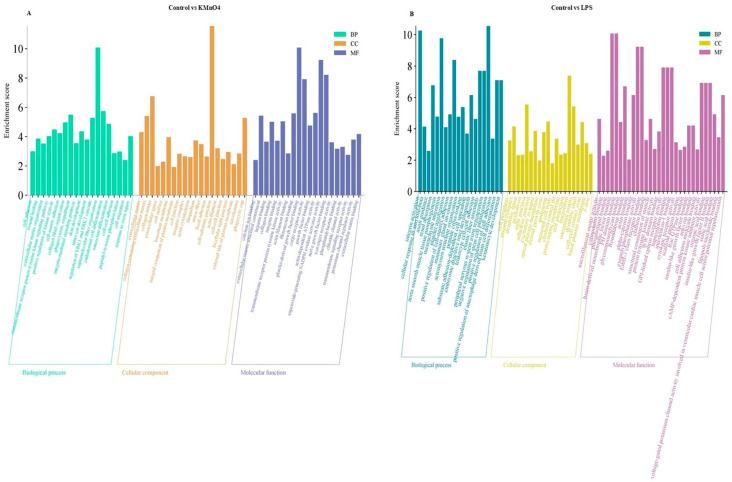
GO Functional Analysis of Differentially Expressed mRNA Enrichment. (**A**) GO Enrichment Analysis of Control vs. KMnO_4_ Group for differentially expressed. The analysis highlights significantly enriched biological processes, cellular components, and molecular functions, indicating the primary functional categories impacted by KMnO_4_ exposure. (**B**) GO enrichment analysis for differentially expressed mRNAs between the control and LPS-treated groups. The enriched GO terms reflect the key biological processes, cellular components, and molecular functions modulated by LPS treatment.

We conducted GO functional enrichment analysis to enhance comprehension of the regulatory function of target genes in oxidative stress, inflammation, and the molecular mechanism underlying apoptosis. These enriched GO terms encompassed 20 biological processes, 20 cellular components, and 20 molecular functions. Based on the findings from the GO annotation analysis, we identified several GO terms associated with inflammation, oxidative stress, apoptosis, negative regulation of glucocorticoid receptor signaling pathway, negative regulation of steroid biosynthesis, metabolic process cell, cytokine receptor activity, and protein transcription factor activity among others (Figure 4A,B).

### 3.6. KEGG Signaling Pathway Enrichment Analysis of Differentially Expressed mRNA

**Figure 5:**
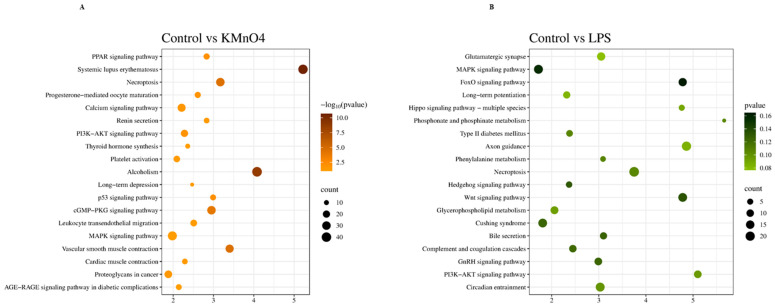
KEGG Signaling Pathway Enrichment Analysis of Differentially Expressed mRNA. (**A**) Kyoto Encyclopedia of Genes and Genomes (KEGG) pathway enrichment analysis of differentially expressed mRNAs between the control and KMnO_4_-treated groups. The pathways enriched in the KMnO_4_ group reveal the signaling cascades and biological systems affected by KMnO_4_-induced oxidative stress and toxicity. (**B**) KEGG pathway enrichment analysis of differentially expressed mRNAs between the control and LPS-treated groups. This analysis uncovers the signaling pathways activated by LPS treatment, providing insight into the inflammatory and immune responses triggered in the uterine tissue.

A KEGG signaling pathway enrichment analysis was conducted on the target genes that exhibited significant differential expressions. The analysis revealed that these targeted genes were enriched in 158 pathways, out of which 78 were enriched in the KMnO_4_ group and 80 in the LPS group. Out of these, 50 were the same in both groups. A total of 20 in the KMnO_4_ group and 20 in the LPS group pathways demonstrated considerable enrichment (Figure 5A,B). Several pathways associated with inflammation and oxidative stress were identified by examining KEGG signaling pathways. These pathways primarily encompass MAPK signaling pathways (RAS, RAF, and ERK1/2), cGMP-PKG signaling pathways (ErAK, VASP, PKA, and CREB), and necroptotic pathways (RIPK1, RIPK3, and MLKL) (Table 3).

### 3.7. Identification of Differentially Expressed LncRNAs of Control vs. KMnO_4_ and Control vs. LPS Groups

**Figure 6:**
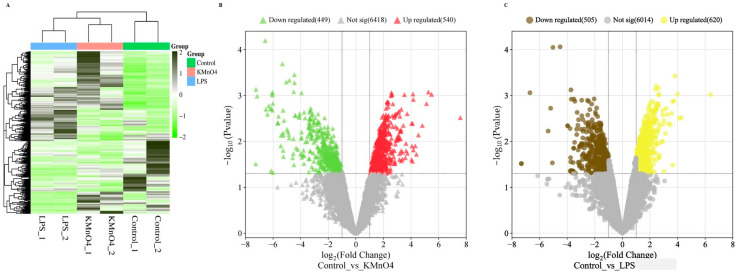
mRNAs and lncRNAs with differential expressions. (**A**) LncRNAs with differential expressions grouped together. Based on FPKMs, hierarchical clustering clusters using log10(FPKM + 1). Genes with higher expressions are indicated in dark green, and those with lower expressions are indicated in light green. (**B**) A volcano plot of transcripts with differential expressions of mRNA. Genes with higher expressions are indicated in red, with lower expressions indicated in green. (**C**) A volcano plot of transcripts with differential expressions of lncRNA. Genes with higher expressions are indicated in yellow, and those with lower expressions are indicated in brown.

In total, 14,546 LncRNAs were acquired, and DESeq2 was used for normalization. A heatmap displayed the expression patterns of these transcripts in various groups. The expression pattern of the KMnO_4_ and LPS groups, as depicted in Figure 6A, differed from that of the control group. The distinctions between the KMnO_4_ and LPS groups were indistinct. This suggests differences in the expressions between the groups treated with KMnO_4_ and LPS. A volcano plot was used to display the expression patterns of these transcripts in the groups treated with KMnO_4_ and LPS. Figure 6B,C labeled the genes based on the −log10 (*p*-value).

### 3.8. Prediction and Functional Analysis of lncRNA Target Genes and mRNAs

**Figure 7:**
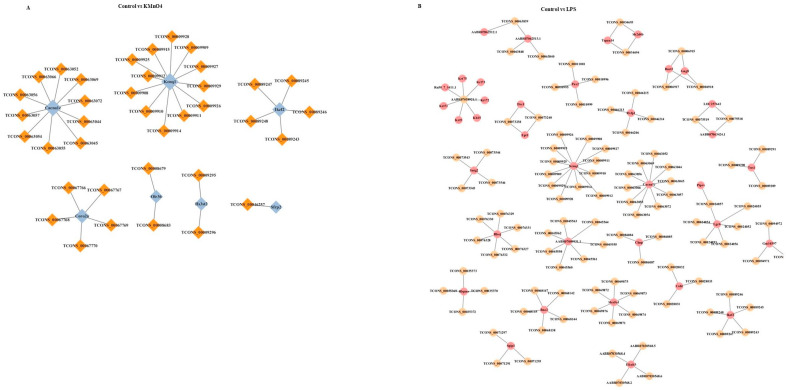
Prediction and Functional Analysis of lncRNA Target Genes and mRNAs. (**A**,**B**) Network diagrams illustrating the interactions between predicted lncRNAs and their target mRNAs in the control group compared with the LPS-treated group (**A**) and the KMnO_4_-treated group (**B**). The visualization highlights key regulatory relationships between lncRNAs and mRNAs, emphasizing the differential gene regulation under treatment conditions.

The mechanism by which lncRNA regulates target genes can be classified into two distinct categories: cis-regulation (occurring within the same genomic region as the target gene) and trans-regulation (occurring in a separate genomic region from the target gene). Predictions were formulated on the regulatory role of differentially expressed lncRNAs on target genes. To facilitate the comprehensive investigation of the activities of lncRNAs in the samples, a network diagram depicting the interaction between differentially expressed lncRNAs and their target genes was constructed. This map serves as a valuable reference and aid in understanding the overall functionality of lncRNAs. Figure 7A shows that Cacnalc targeted TCONS_00063069, TCONS_00063072,TCONS_00063042,TCONS_00063045,TCONS_00063055,TCONS_00063054, TCONS_00063055, TCONS_00063057, TCONS_00063056, and TCONS_00063066.

On the other hand, IKzf2 targeted TCONS_00089245, TCONS_00089246, TCONS_00089243, TCONS_00089248, and TCONS_00089247 in KMnO_4_ group. In the LPS group, Pax2 targeted the transcripts TCONS_00010999, TCONS_00010996, TCONS_00010995, TCONS_00011001, and the gene Slc7a7, are known to interact with the transcripts TCONS_00030709, TCONS_00030719, TCONS_00030721, and TCONS_00030713 (Figure 7B).

### 3.9. Analysis of lncRNA Target Genes via GO Enrichment and KEGG Enrichment

**Figure 8:**
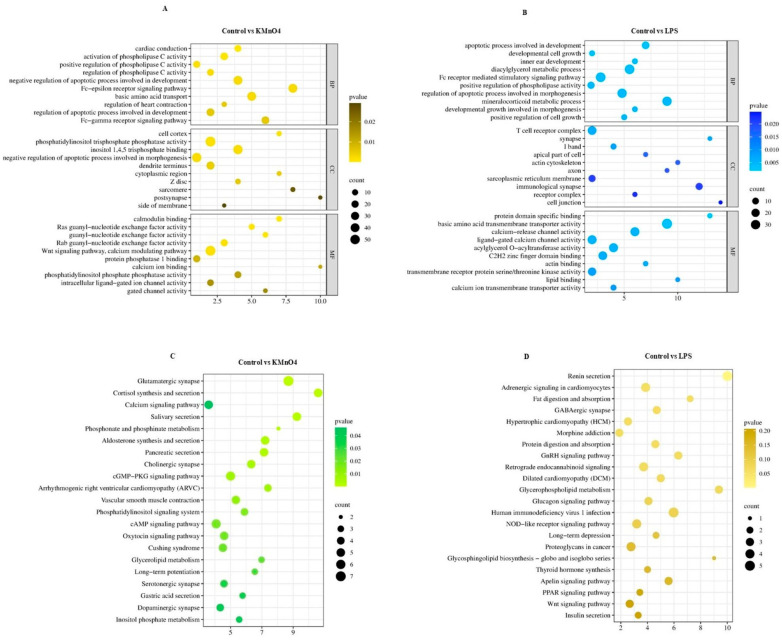
Analysis of lncRNA Target Genes via GO and KEGG Enrichment. (**A**,**B**) These panels present the GO functional enrichment analyses of lncRNA target genes. The comparisons of control vs. KMnO_4_ (**A**) and control vs. LPS (**B**) reveal biological processes, cellular components, and molecular functions. (**C**,**D**) The bubble bar plots depict the most enriched KEGG pathways in the comparison of control vs. KMnO_4_ (**C**) and control vs. LPS (**D**). The bubble size represents the gene count, while the color intensity indicates the significance of enrichment.

A GO analysis was performed on the target gene, using a significance threshold of *p* < 0.05 to determine enrichment in GO annotations. The GO concepts for biological processes, cellular components, and molecular functions are presented in Figure 8A,B, each displaying 10 GO terms. The target gene underwent KEGG analysis, with a significance threshold of *p* < 0.05 used to determine the enrichment of the KEGG pathway and obtain the analysis results. The results of the KEGG pathway analysis are presented in the bubble diagram of the KEGG pathway, as shown in Figure 8C,D. The KEGG enrichment analysis identified target genes linked to oxidative stress and inflammation that are involved in pathways such as the Wnt signaling system and the cGMP-PKG signaling pathway. Furthermore, the cAMP signaling pathway and the GnRH signaling system involved in hormonal regulation were also recognized.

### 3.10. Validation of Genes by RT-qPCR

**Figure 9:**
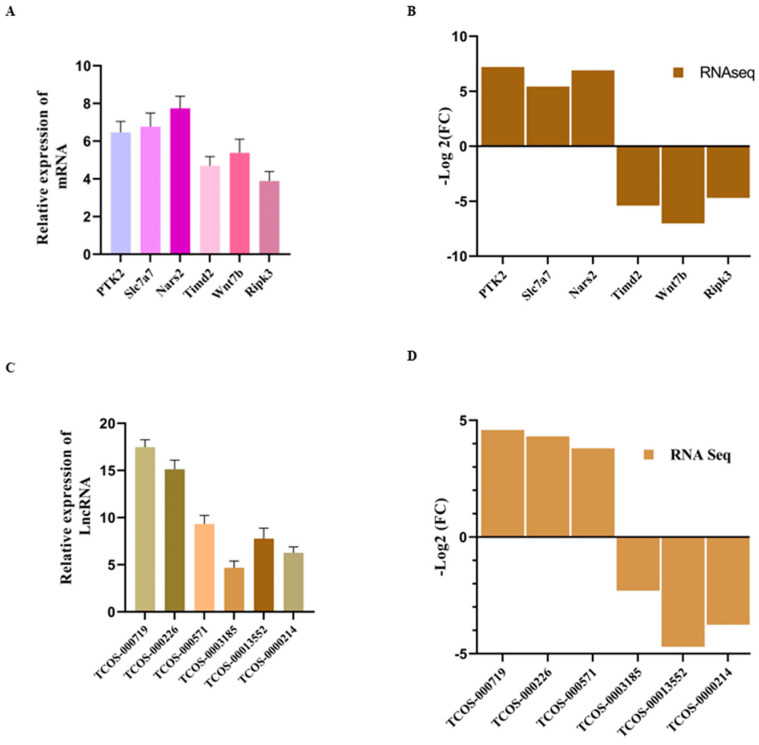
RT-qPCR Validation of RNA-Seq Results. (**A**,**B**) Bar plots comparing the expression levels of upregulated (**A**) and downregulated (**B**) mRNAs, as determined by RNA-Seq and validated through RT-qPCR. The consistent trends between the qPCR and RNA-Seq results confirm the reliability of these transcriptomic data. (**C**,**D**) Bar plots showing the differential expression of selected lncRNAs, comparing RNA-Seq data with RT-qPCR validation. Upregulated (**C**) and downregulated (**D**) lncRNAs exhibit consistent expression patterns across both methods.

To validate the precision of the sequencing process, this study opted to perform quantitative PCR verification on a subset of differentially expressed mRNAs and lncRNAs. We picked shared mRNA and lncRNAs from both groups (Control vs. KMnO_4_; Control vs. LPS). The findings are depicted in Figure 9A–D. qPCR findings exhibit a high degree of concordance with the sequencing outcomes, corroborating the accuracy and reliability of these sequencing data.

## 4. Discussion

Uterine bacterial infections are frequently observed in multiparous cows, causing notable uterine destruction, decreased productivity, and lowered fertility. One of the key pathogens in these infections is the endotoxin *Escherichia coli* (*E. coli*), which is also linked to inflammatory disorders such as endometritis and acute lung injury [7,29]. Although inflammation is a protective response aimed at fighting infections, excessive or prolonged inflammation can lead to tissue damage, organ dysfunction, and even life-threatening conditions [30,31]. Clinically, antibiotics are the primary treatment for uterine infections, but their overuse comes with significant drawbacks, including negative effects on reproductive health and suppression of immunity in dairy cows [32,33]. Additionally, the global rise of antibiotic resistance has become a critical concern [34,35].

An alternative treatment, KMnO_4_, has long been used because of its strong oxidizing properties that make it effective in treating bacterial and fungal infections [36]; however, while KMnO_4_ is useful in wound disinfection, its oxidizing potential can also cause significant damage to healthy tissues. In cattle, excessive KMnO_4_ exposure has been associated with ulcers and other tissue injuries. This dual-edged nature of KMnO_4_ underscores the need to understand its biological effects more thoroughly, especially in sensitive tissues such as the uterus.

In this study, we utilized a rat model to compare the effects of KMnO_4_ and LPS on uterine tissues, focusing on inflammation and oxidative stress at the molecular level. LPS is a well-established inducer of inflammation and oxidative damage, commonly used as a model for reproductive disorders [37]. The transcriptional profiles of KMnO_4_-treated rats may differ significantly from those exposed to other common antiseptics used in veterinary medicine, such as iodine-based solutions or chlorhexidine. Each antiseptic has a unique mechanism of action, leading to distinct patterns of gene expression and molecular damage [8]. We sought to investigate the transcriptional changes, specifically in lncRNAs, which play a crucial role in gene regulation and could potentially serve as therapeutic targets.

Histopathology was employed in the present study to ascertain the presence of oxidative stress inside the uterine tissues of rats. Necrosis, edema, and leukocyte infiltration were observed through direct observation. Furthermore, the results of the wet and dry weight of uterus tissues also show obvious change. Moreover, biochemical assays measuring MDA, SOD, and MPO further confirm the successful establishment of the animal model. In addition to assessing the effects of KMnO_4_ and LPS within the uterine environment, we collected lung tissues for histopathological examination; however, our findings indicated no significant alterations in these tissues. Furthermore, biochemical analyses were conducted, revealing that the toxicity associated with LPS and KMnO_4_ was confined to uterine tissues. Thus, our results substantiate the successful establishment of our experimental model.

Following the extraction of total uterine RNA, high-throughput sequencing was conducted on the Illumina platform to elucidate the profiles of long non-coding RNA (lncRNA) and gene expression in KMnO_4_ treated, comparing LPS group in rat uterine tissues.

LncRNAs are a class of RNA molecules with therapeutic capabilities because of their ability to induce alterations in DNA transcription through methylation and acetylation processes [38]. This work examined the mRNA and lncRNA expression in tissues subjected to oxidative stress. A total of 1649 differentially expressed messenger RNAs (mRNAs) were found in the KMnO_4_-treatment group, and 1383 were found in the LPS-treatment group by screening. In addition, 1125 expressed lncRNAs were found in the KMnO_4_-treatment group, and 989 were found in the LPS-treatment group. The function enrichment analysis revealed that a considerable proportion of the genes that exhibited differential expression were implicated in several biological processes, including reactive oxygen species, signal transduction involved in gene expression regulation, Wnt signaling pathway, regulation of macrophage activation, and various biochemical metabolic processes. The enrichment analysis of the KEGG pathway revealed that the differentially expressed genes primarily engaged in signal pathways associated with the MAPK signaling pathway, cGMP-PKG pathway, Wnt pathway, Necroptosis, and cAMP signaling pathways. In addition to genes exhibiting variable expression, most lncRNAs had an ambiguous functional role. The hypothesis in this study posited that lncRNA has a regulatory role in gene expression, as mentioned earlier. Consequently, the function of lncRNA was predicted by considering its associated coding genes, as indicated by previous research [39].

This study estimated target genes of differentially expressed lncRNA using cis and trans methods. Subsequently, GO and KEGG studies were conducted on the identified target genes to explore the potential functional role of the lncRNA [40,41]. Through the utilization of enrichment analysis of GO and KEGG pathways, as well as coding-noncoding co-expression network analysis, we have determined that the differentially expressed target genes primarily exhibit associations with inflammation and apoptotic signaling pathways.

The lncRNAs TCONS_00030719, TCOS_00078464, and TCONS_00082446 exhibited differential expressions in the Control vs. KMnO_4_ group. They were enriched in various signaling pathways. The signaling pathways strongly correlate with oxidative damage and inflammation. The identified gene Slc7a7 targets TCONS_00030719, TCONS_00030709, TCONS_00030721, and TCONS_00030713. The gene Slc7a7 was found to be implicated in the regulation of lifespan, as well as in the cAMP signaling route and calcium signaling pathway, according to the KEGG pathway enrichment analysis. Prior research has established a strong association between the Slc7a7 gene and human longevity and hypertension. This gene plays a crucial role in regulating the immune response by activating transcription factors, which influence gene expression in immune cells [42,43].

Furthermore, in Control vs. LPS, the target lncRNAs predicted for LGR6 include TCONS_00024057, TCONS_00024055, TCONS_00024054, TCONS_00024052, TCONS_00024053, and TCONS_00024056. GPRs that are broadly expressed in a variety of cells, including neutrophils, monocytes, cochlear hair cells, and astrocytes, also contain different types, such as leucine-rich repeat-containing G protein-coupled receptor 6 (LGR6) [44,45,46]. According to the literature, it has been documented that LGR6 can modulate many inflammatory pathways and contribute to generating reactive oxygen species in the context of oxidative stress [47,48]. According to recent research, in a rat model of subarachnoid hemorrhage, Maresin 1 activates LGR6 to reduce neuroinflammation via the CREB/JMJD3/IRF4 pathway [49]. Maresin 1 reduces diabetic kidney disease by activating the cAMP-SOD2-ROS pathway mediated by LGR6 in another study [48].

In summary, this study provides new insights into the molecular mechanisms underlying KMnO_4_ and LPS-induced uterine damage, particularly the role of lncRNAs in regulating inflammation and oxidative stress. Our findings reveal that lncRNAs, through their interactions with key genes such as Slc7a7 and LGR6, may act as important regulators of the immune response in reproductive tissues. Understanding these regulatory networks could pave the way for the development of targeted molecular therapies to treat uterine.

## Figures and Tables

**Table 1:** qPCR Primers for LncRNAs and mRNAs.

Name	Accession Number	Product Size (bp)	Primer Sequence (5′-3′)
PTK2	NM_013081.2	246	Forward:CGTGTGGATGTTTGGTGTGT Reverse:TGCACCTTCTCTTCCTCCAG
Slc7a7	NM_031341.2	190	Forward:CCTGTTCTTCCCCATCGTCT Reverse:TGTGGTAGACGCTACGATCC
Nars2	NM_001034921.2	235	Forward:GTGGATCCGGTCAGTCAGAT Reverse:AAATGCCTTGGCTTCACAGG
Timd2	NM_001013855.1	223	Forward:ACTGAAGCAATCCCTCCACA Reverse:CTTCAATTCTGGCCTGCTCC
Wnt7b	NM_001009695.2	161	Forward:TTCACAACAATGAGGCAGGC Reverse:AGCTGCGTTGTACTTCTCCT
RIPK3	NM_139342.2	182	Forward:TCCACATTTCAGGGAGGGTC Reverse:ACCACCTCAGCTTCTCTTCC
TCOS_000719	-	216	Forward:AACAAGGAACAAGGCCACAC Reverse:GGCTTTCCCAGGTCTTAGGT
TCOS_000226	-	234	Forward:CTGTCTGAAGGGCACATGTG Reverse:TCCAGGATCTCAGCCACAAA
TCOS_000571	-	197	Forward:GCTGGATGTTGAGAGCACAG Reverse:TTCTAGCTCCCCACTCCTCT
TCOS_0003185	-	250	Forward:ACTGTGTGTGCTGGGTATGA Reverse:TGGCAAGTTTCGACTGTGTG
TCOS_13552	-	207	Forward: ACAACACTACCAGGGGACAG Reverse:GCAGAGGCCCATAGTCTAGG
TCOS_000214	-	201	Forward:GTTCCCAAGGCTTGACCCAA Reverse:AAGAATTGCCTGGTGTGTCCT

**Table 2:** Summary of Reads.

Sample Name	Clean Reads	Clean GC (%)	Mapped Reads (%)	Effective Rate (%)
Control_1	57,300,644	50.09	93.54	87.53
Control_2	51,742,860	50.69	91.38	85.51
KMnO_4__1	57,128,986	49.36	94.43	88.36
KMnO_4__2	51,422,446	50.56	93.09	88.63
LPS	65,824,496	49.65	92.68	85.74
LPS	78,704,690	49.41	92.91	87.48

**Table 3:** KEGG pathways and their Genes.

KEGG Pathways	Enrichment	Genes
MAPK signaling pathway	1.98	Rac3; Cacnla; Cacnalc; Mef2c; Dusp8
cGMP-PKG signaling pathway	2.95	Wos3; Kcnj8; Pde3a; Mylk; Kcnmal; Mef2c; Pde5a; Kcnmb1
Necroptosis	3.17	RIPK1; RIPK3; MLKL; H2afx
FOXO signaling pathway	4.78	Cdkn2b; Tgfb2; Pck1; Fbxo32; PlKI; Ccdn2; Ccnb1; Irs2; Slc2a4
GnRH pathway	2.99	Plcb1; Cacnalc; Plcb4; Adcy3; Calml3; Camk2g; Adcy9
Wnt pathway	4	SfrD2; Plcbl; Rspo3; Camk2g; Nfatc4; Rac3; Sfrp4; Ccdn2; Plcb4
PI3K-AKT signaling pathway	5.1	PI3K; PTEN; AKT; FOXO; CytokineR
P53 signaling pathway	2.99	CHK2; P53; Fas; Caspase8
TNF signaling pathway	2.21	TNFR1; TRADD; TRAF; TAK1; TAB1/2; MKK; IL18R
JAK-STAT signaling pathway	2.38	JAK; STAT; IL13ra; IL6r; IL11r; IL4r

## Data Availability

Raw data supporting the conclusions of this article will be made available by the authors upon request.
